# Draft Genome Sequence of *Hymenobacter* sp. Strain AT01-02, Isolated from a Surface Soil Sample in the Atacama Desert, Chile

**DOI:** 10.1128/genomeA.01701-15

**Published:** 2016-02-11

**Authors:** Anders Cai Holm Hansen, Ivan Glaucio Paulino-Lima, Kosuke Fujishima, Lynn Justine Rothschild, Peter Ruhdal Jensen

**Affiliations:** a Microbial Biotechnology & Biorefining, National Food Institute, Technical University of Denmark, Lyngby, Denmark; b NASA Postdoctoral Program Fellow, NASA Ames Research Center, Moffett Field, California, USA; c University Affiliated Research Center, NASA Ames Research Center, Moffett Field, California, USA; d NASA Ames Research Center, Moffett Field, California, USA

## Abstract

Here, we report the 5.09-Mb draft genome sequence of *Hymenobacter* sp. strain AT01-02, which was isolated from a surface soil sample in the Atacama Desert, Chile. The isolate is extremely resistant to UV-C radiation and is able to accumulate high intracellular levels of Mn/Fe.

## GENOME ANNOUNCEMENT

*Hymenobacter* sp. strain AT01-02, isolated from the Atacama Desert, Chile, is a Gram-negative pink-pigmented bacterium that thrives in an extremely dry environment, where it is exposed to large temperature variations and high levels of solar UV radiation (1, 2). Several *Hymenobacter* spp. were reported to be radiation resistant (3–6), and isolate AT01-02 is more resistant than *Deinococcus radiodurans* to UV-C irradiation, being able to accumulate high intracellular levels of Mn/Fe (our unpublished data).

Here, we describe a draft genome sequence of *Hymenobacter* sp. AT01-02 to investigate the genetic mechanisms involved in the survival of this organism in such an extreme environment. Total genomic DNA was extracted from colonies using the Microbial DNA isolation kit (Mo Bio Laboratories, Solano Beach, CA, USA), according to the manufacturer’s instructions. The genome was sequenced on an Ion Torrent instrument, producing 2,680,302 reads, which were trimmed with respect to quality and size (cutoff, Q20; size, 50 to 270 bp). The trimmed reads were *de novo* assembled using CLC bio Genomics Workbench into 272 contigs, constituting a total genome size of 5,043,991 bp, with sizes ranging from 517 bp to 315,336 bp. The average size was 18,544 bp, and the *N*_50_ was 74,558 bp. The G+C content was 55.5%, with a genome coverage of 90×.

We annotated the assembled genome using the IMG-ER portal (https://img.jgi.doe.gov/cgi-bin/er/main.cgi). We found 50 predicted RNAs, including 4 rRNAs, 42 tRNAs, and 4 miscellaneous RNAs. The RAST result showed 4,821 protein-coding genes (coding sequences), including 1,360 known and 3,461 unknown subsystems, with 759 protein-coding genes connected to KEGG pathways.

We found 35 genes related to stress response, including 24 genes for oxidative stress, 5 genes for cold shock, 5 genes for general stress, and 1 gene for osmotic stress. We also found 21 genes related to the biosynthesis of secondary metabolites, 14 genes related to drug resistance, and 1 multidrug resistance gene (efflux pump membrane protein). Additionally, we identified 38 genes for the metabolism of terpenoids and polyketides, 29 genes for xenobiotic biodegradation and metabolism, 43 genes related to DNA replication and repair (UvrABC system, *recA*, and MutS), and 29 genes related to membrane transport (including 16 ABC transporters, 11 secretory systems, and 2 manganese transporters of the natural resistance-associated macrophage protein [NRAMP] family). Similar to what was reported recently for a different *Hymenobacter* isolate (7), we found 4 teichoic and 3 lipoteichoic biosynthesis genes, which are characteristically present in Gram-positive organisms, although AT01-02 is Gram negative. Interestingly, 16 genes indicated the presence of bacteriophage (including 9 integrase, 5 transposase, 1 terminase, and 1 lamin tail domain), which is related to low optical densities obtained with liquid cultures, and to a clear center observed in old colonies grown on agar plates. Also, 2 genes were related to exopolysaccharide biosynthesis, which is connected to morphological aspects of the colonies grown on plates, including brilliance and stickiness.

The genome of AT01-02 exhibits a diverse suite of stress-responsive and pigment-producing genes, along with bacteriophage genes and many genes for the biosynthesis of secondary metabolites and biodegradation of xenobiotics. These characteristics represent its potential for industrial applications and will provide insights to better understand the survival mechanisms against environmental stresses, such as desiccation and UV radiation.

### Nucleotide sequence accession numbers.

This whole-genome shotgun project has been deposited at DDBJ/EMBL/GenBank under the accession number JZIR00000000. The version described in this paper is version JZIR02000000.
